# Exacerbation predictive modelling using real-world data from the myCOPD app

**DOI:** 10.1016/j.heliyon.2024.e31201

**Published:** 2024-05-14

**Authors:** Henry M.G. Glyde, Alison M. Blythin, Tom M.A. Wilkinson, Ian T. Nabney, James W. Dodd

**Affiliations:** a EPSRC Centre for Doctoral Training in Digital Health and Care, University of Bristol, Bristol, UK; b My mHealth, Bournemouth, UK; c My mHealth and Clinical and Experimental Science, University of Southampton, Southampton, UK; d School of Engineering Mathematics and Technology, University of Bristol, Bristol, UK; e Academic Respiratory Unit, Translational Health Sciences, Bristol Medical School, University of Bristol, Bristol, UK

**Keywords:** Chronic obstructive pulmonary disease (COPD), mHealth, Machine learning, Prediction models

## Abstract

**Background:**

Acute exacerbations of COPD (AECOPD) are episodes of breathlessness, cough and sputum which are associated with the risk of hospitalisation, progressive lung function decline and death. They are often missed or diagnosed late. Accurate timely intervention can improve these poor outcomes. Digital tools can be used to capture symptoms and other clinical data in COPD. This study aims to apply machine learning to the largest available real-world digital dataset to develop AECOPD Prediction tools which could be used to support early intervention and improve clinical outcomes.

**Objective:**

To create and validate a machine learning predictive model that forecasts exacerbations of COPD 1–8 days in advance. The model is based on routine patient-entered data from myCOPD self-management app.

**Method:**

Adaptations of the AdaBoost algorithm were employed as machine learning approaches. The dataset included 506 patients users between 2017 and 2021. 55,066 app records were available for stable COPD event labels and 1263 records of AECOPD event labels. The data used for training the model included COPD assessment test (CAT) scores, symptom scores, smoking history, and previous exacerbation frequency. All exacerbation records used in the model were confined to the 1–8 days preceding a self-reported exacerbation event.

**Results:**

TheEasyEnsemble Classifier resulted in a Sensitivity of 67.0 % and a Specificity of 65 % with a positive predictive value (PPV) of 5.0 % and a negative predictive value (NPV) of 98.9 %. An AdaBoost model with a cost-sensitive decision tree resulted in a a Sensitivity of 35.0 % and a Specificity of 89.0 % with a PPV of 7.08 % and NPV of 98.3 %.

**Conclusion:**

This preliminary analysis demonstrates that machine learning approaches to real-world data from a widely deployed digital therapeutic has the potential to predict AECOPD and can be used to confidently exclude the risk of exacerbations of COPD within the next 8 days.

## 1 Introduction

Affecting an estimated 391·9 million people aged over 30years [1], chronic obstructive pulmonary disease (COPD) is the 3rd leading cause of death and disability worldwide [[2], [3], [4]]. People living with COPD are at risk of episodes of sustained worsening of respiratory symptoms (breathlessness, sputum volume, and sputum purulence) beyond their usual stable state referred to as acute exacerbations. An exacerbation at the time of this analysis was defined by GOLD as an acute worsening of respiratory symptoms that results in additional therapy. Exacerbations of COPD are associated with significant economic costs [[5], [6], [7]], risk of death, rapid decline in lung function, and impaired quality of life [[8], [9], [10], [11]]. Recently, a research priority-setting partnership in COPD found the highest-rated problem by patients was to better ‘identify better ways to prevent exacerbations’ [12].

Wilkinson et al. demonstrated that preventing delay in treating an exacerbation (with oral steroids and antibiotics) was associated with a reduced recovery time [13]. This has led to efforts to monitor and track patients with COPD to identify and treat exacerbations early. Some successful examples include that of Dinesen and colleagues who used telehealth monitors to collect data including blood pressure, pulse, weight, oxygen saturation (SpO2) and lung function (spirometry) monitored by healthcare professionals (GP, nurse, or doctor) [14]. Exacerbations and/or specific symptom cutoff points were not pre-defined; instead, the monitoring healthcare professional evaluated patient data and initiated contact with the patient for an appropriate intervention based on their individual circumstances. This system managed to significantly reduce the mean hospitalisation rate by greater than half. Furthermore, Calvo et al. used remote monitoring (blood pressure, oxygen saturation and heart rate daily, and peak expiratory flow (PEF) three times a week) to detect and intervene at the earliest stages of exacerbations [15]. A clinical alert was generated if measurements exceeded pre-established limits, e.g. heart rate surpassing 100 beats per minute or dropping below 50 beats per minute, an SpO2 decrease of approximately 3 % from the individual patient's limit, a systolic pressure rise of nearly 30 mmHg or a drop of 30 mmHg, and a peak flow reduction exceeding 70 % of the personal benchmark. Subsequently, a nurse contacted the patient to administer a series of clinical questionnaires, confirming their symptoms and provide medical recommendations to the patient regarding COPD exacerbations, This resulted in a significant reduction in emergency department visits (20 in the intervention vs 57 in conventional care), hospitalisations (12 vs 33), duration of hospital stay (105 vs 276 days) and need for non-invasive mechanical ventilation (0 vs 8), and time to the first severe exacerbation (141 vs 77) [15]. However, these approaches are both burdensome to both patient and resource intensive for the clinician limiting widespread uptake. In addition, Orchard and colleagues found such algorithms often have very low accuracy with some performing no better than chance [16]. This has led to increased interest in the possibilities of automated systems offered through digital platforms and AI.

We aimed to use machine learning, a subset of artificial intelligence, to analyse real world routine patient entered data a widely used digital COPD self-management app, myCOPD app. myCOPD, was developed as an interactive cloud-based digital self-management tool, which is widely used within the NHS. For patients, myCOPD serves as a comprehensive platform for entering and storing health-related data, enabling the tracking of health progress, including daily symptom monitoring and health status. The app includes a self-management plan with a checklist and target options. Furthermore, it provides access to current health and lifestyle information through educational videos, encouraging exercise with approved pulmonary rehabilitation courses. Patients can conveniently record their medication in the medication diary and set reminders.

## 2 Methodology

### 2.1 Data extraction and preparation

The myCOPD patient entered data was extracted on December 7, 2021, and contained 13682 activated patient users (users who have activated their account and logged into the app). The initial stage involved data cleaning; identifying and rectifying errors in the dataset to ensure its suitability for analysis. Following the data cleaning process, the dataset underwent pre-processing using an algorithm designed to extract entries available 14 days before each data point. A 14-day timeframe was chosen based on clinical guidance, suggesting that respiratory symptoms are likely to develop up to 14 days before an exacerbation occurs. We then conducted co-creation workshops between engineers and clinicians to define an exacerbation label based on self-reported symptom scores. The exacerbation label was defined as 14-days of no exacerbation before a report of a symptom score of 3 “moderate deterioration” (they have taken antibiotics and/or corticosteroids) or 4 “severe deterioration” (they have contacted primary care provider, called 999, or have been hospitalised). The stable label was defined as 14-days of no exacerbation before a report of a symptom score of 1 “normal for me” (no change in normal symptoms) or 2 “mild deterioration” (have used their rescue inhaler).

We selected and processed 12 features from myCOPD patient user data and attached them to our labels for modelling. These features encompass a comprehensive set of stable and dynamic variables, including health status (COPD Assessment Score – CAT), symptom score, age, gender, disease severity (GOLD stage), breathlessness score (mMRC dyspnea), smoking history (pack years), and medication usage including antibiotics and steroids in addition to hospitalisations, and exacerbation type (severity of last exacerbation).

The CAT score, derived from the COPD Assessment Test, is a validated health status tool using 8 graded questions to assess the impact of COPD on daily living. Users are prompted to complete the CAT monthly but can do so as frequently as they prefer within the app. We developed a mean stable CAT feature which was generated by calculating the average CAT score when users reported their symptoms as "normal for me”. We then created a change in CAT feature which is determined by subtracting the CAT mean from their specific CAT score for that instance. The symptom score is a 4-point variable reflecting the patient's daily well-being, and users are encouraged to complete it daily. Notably, a rise in the symptom score from 1 to 2 before an exacerbation may indicate the use of a reliever inhaler, suggesting a decline in symptoms and the potential onset of an exacerbation. Age is recorded as the individual's age at the time of data entry, and gender is self-reported as male, female, or not specified. Fort further details on dynamic and stable variables see supplement.

When this analysis was conducted for this study the GOLD (Global Initiative for Chronic Obstructive Lung Disease) classification used for categorizing the severity of COPD was as follows:

GOLD A/1 (Mild): FEV1 is equal to or greater than 80 % of the predicted value.

GOLD B/2 (Moderate): FEV1 is between 50 % and 79 % of the predicted value.

GOLD C/3 (Severe): FEV1 is between 30 % and 49 % of the predicted value.

GOLD D/4 (Very Severe): FEV1 is less than 30 % of the predicted value or FEV1 is less than 50 % with chronic respiratory failure.

Participants were excluded from the sample for not having data that matched either the exacerbation or the stable label (n = 7457) or if they were missing any one of the 12 features (n = 3998). We chose not to impute missing data and instead opted for the removal of participants or data points with incomplete information. This decision was driven by the aim to maintain real-world representativeness and avoid reliance on assumptions. Lastly, we removed myCOPD users who had not reported at least one exacerbation that matched our label to ensure the sample only included patients who experienced both a stable state and an exacerbation state (n = 1721). This is because the current modelling approach is focused on patient users who are highly engaged in the app and use the system for support when they are stable and when they are exacerbating. This resulted in 506 myCOPD patient users with 55,066 stable state records and 1263 exacerbation state records. Details of the characteristics of our study population are included in Table 1.

**Table 1:** Baseline characteristics of study participants.

Characteristic	Participants (n = 506)
Age in years (mean, SD)	68.5 (10.2)
Sex (male, female, not available)	221, 193, 92
GOLD classification of severity of airflow limitation (number of participants)	
A	44
B	137
C	51
D	274
mMRC dyspnea score (number of participants)	
0	42
1	150
2	167
3	86
4	61
Smoking status (number of participants)	
Never smoked	14
Ex-smoker	440
Current smoker	52
Pack year (median, IQR)	30 (27)
Number of hospital admissions in the previous year (median, IQR)	0 (0)
Number of rescue packs in the previous year (median, IQR)	2 (2.5)

GOLD = Global Initiative for Chronic Obstructive Lung Disease, mMRC=Modified Medical Research Council.

**Fig. 1:**
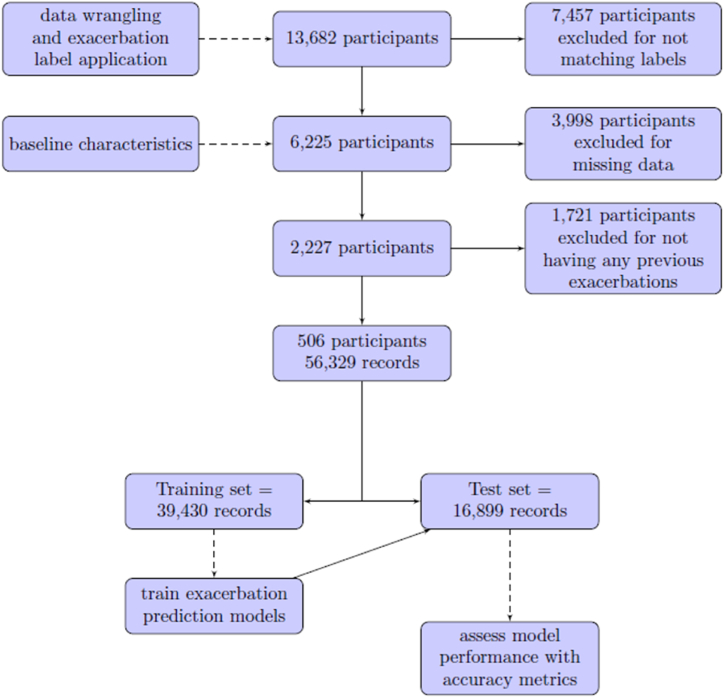
Participant selection and study design. A total of 506 participants with 56,329 records in myCOPD had their data randomly split into training (records = 39,430) and testing (records = 16,899) sets. The EasyEnsemble classifier algorithm and an AdaBoost with a cost-sensitive tree algorithm were applied to the training dataset. Both models were tested in the testing set of the myCOPD sample.

After labelling and combing the patient data, we proceeded to train and validate our machine learning algorithms on the sample data to produce exacerbation prediction models. We created two training and test sets to validate our models. In the first set, data from all participants were randomly assigned to training and test sets, as illustrated in Fig. 1. For the second test set, participants were randomly divided into training (n = 354) and testing (n = 152), encompassing 38,932 and 17,397 records, respectively.

### 2.2 Classifier design

Within the myCOPD sample selected for modelling, there is a large class imbalance between the exacerbation and stable labels (a ratio of 1:44). We applied two algorithms designed to train models on imbalanced datasets. The first, AdaBoost classifier with a modified weak learner, is a meta-estimator that fits a weak learner (a classification algorithm that performs slightly better than random chance) on the original dataset and then fits additional copies of the learner on the same dataset [17]. This puts more weight on difficult-to-classify instances and less on correct classifications. The weak classifier (also called the weak learner) is a decision tree with a single split commonly known as a decision stump. Instead of the default decision stump used in AdaBoost, we used the Decision Tree Classifier (DTC) from the scikit-learn package [18]. We modified the class weights of the DTC by tuning with grid search on our training set for hyperparameter optimisation. This modification of the DTC is often referred to as a cost-sensitive tree (CST). Upon optimisation of the class weights, the CST was applied as the weak learner of the AdaBoost algorithm and run on the training set.

The second algorithm used for modelling is the EasyEnsemble Classifier from the imbalanced-learn package [19]. The EasyEnsemble Classifier is an ensemble of AdaBoost learners trained on different balanced bootstrap samples. It works by randomly undersampling the majority class from the original dataset every time a decision stump is trained. This means the ensemble prediction model is trained on balanced classes but has sampled many cases from the majority class, so the final model has more knowledge of the entire dataset.

## 3 Results

Demographic characteristics of participants, markers of disease severity (including admissions in the previous year), and smoking status are displayed in Table 1. The participants in the modelling sample have later-stage/more severe COPD with study participants falling into the GOLD stage D and the average number of rescue packs used by participants in the previous year was 2.28.

### 3.1 Development of an exacerbation prediction model

**Fig. 2:**
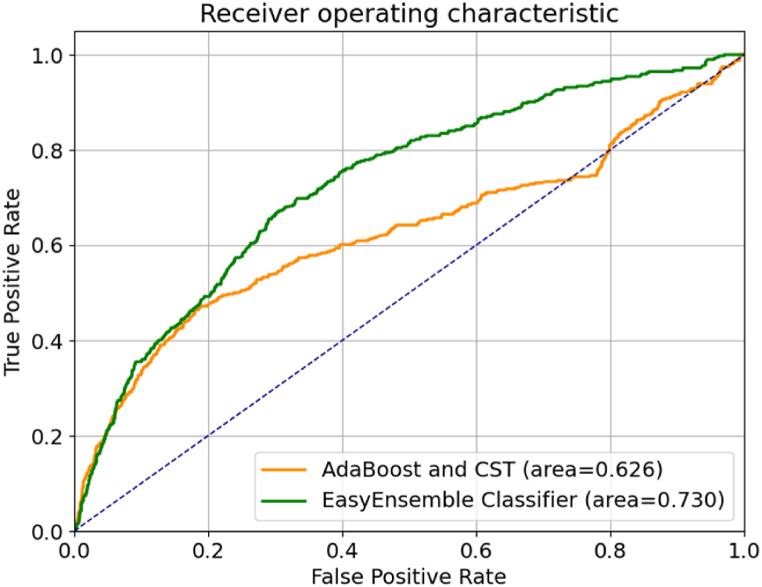
Receiver Operating Characteristic (ROC) Curves comparing The EasyEnsemble classifier model with the AdaBoost with a cost-sensitive tree model. Models were trained in 70 % and tested in 30 % of the myCOPD sample.

We used AUROC (Area Under the Receiver Operating Characteristic) curves to graphically represent the trade-off between sensitivity and specificity at various decision thresholds, providing a comprehensive evaluation of our model's performance. The AUROC curves depicting the performance of the two models, where the data was randomly divided into training and test sets, are presented in Fig. 2. In this analysis some participant data are present in both the training and test sets. Notably, there exists a significant contrast in the AUROC between the EasyEnsemble Classifier (0.730) and the AdaBoost with CST (0.626). Much of the improvement seems to be at a decision threshold leading to higher false positive rates. Taken together, these findings indicate that the EasyEnsemble Classifier outperforms the AdaBoost with CST as a predictive model for our dataset.

**Fig. 3:**
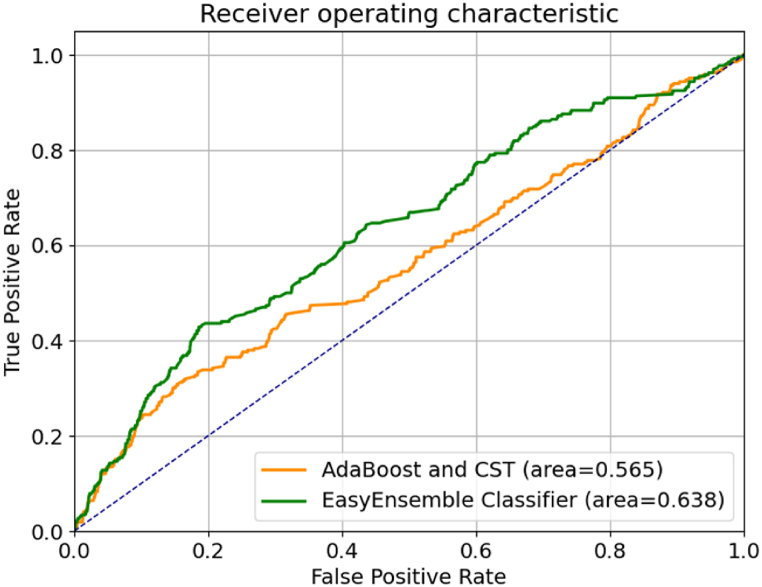
ROC curves comparing the EasyEnsemble classifier model with the AdaBoost with a cost-sensitive tree model. Models were trained on data from 354 participants and tested on data from 152 participants of the myCOPD sample.

The AUROC curves illustrating the performance of the two models, with participants divided into training (n = 354) and test (n = 152) sets, are displayed in Fig. 3. Notably, a reduction in the Area Under the Curve (AUC) is observed, with the EasyEnsemble Classifier scoring 0.659 compared to the AdaBoost with CST at 0.564. It's important to highlight that there is a decrease in AUC for both models when the split is based on participants, suggesting potential challenges in inter-patient generalisation for these models.

**Fig. 4:**
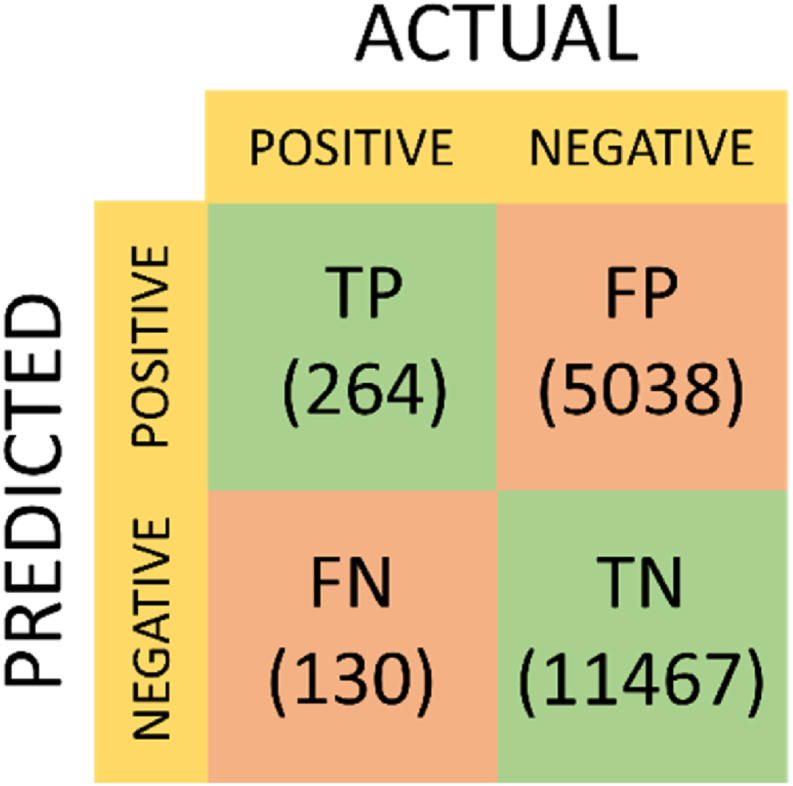
Confusion Matrix depicting the performance of the EasyEnsemble Classifier. The matrix includes True Positives (TP), False Positives (FP), False Negatives (FN), and True Negatives (TN). 5038 instances of stable health reports were misclassified as exacerbations, while 130 exacerbations were misclassified as stable health.

**Fig. 5:**
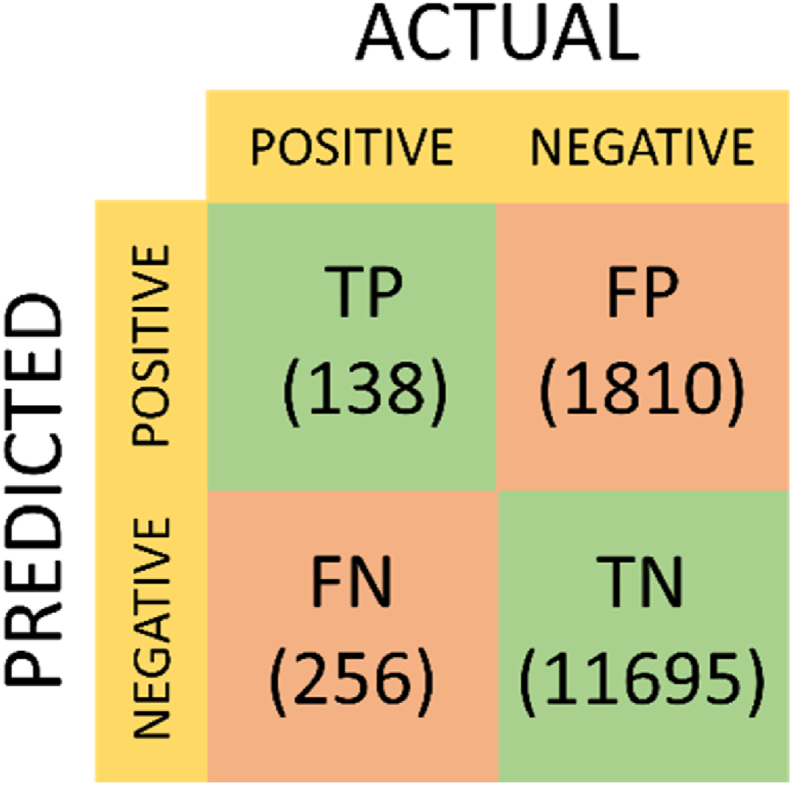
Confusion Matrix depicting the performance of the AdaBoost with CST. The matrix includes True Positives (TP), False Positives (FP), False Negatives (FN), and True Negatives (TN). 1810 instances of stable health reports were misclassified as exacerbations, while 256 exacerbations were misclassified as stable health.

The performance of the two models, with data randomly split into training and test sets, is presented through confusion matrices in Fig. 4, Fig. 5. Examining the confusion matrix for the EasyEnsemble Classifier reveals a notable number of false positives, particularly when compared to the outcomes of the AdaBoost with CST. Conversely, the AdaBoost with CST exhibits a significantly higher number of false negatives. Additional metrics such as sensitivity, specificity, positive predictive value (PPV), and negative predictive value (NPV) are detailed in Table 2. While the ROC curves in Fig. 2 suggest that the EasyEnsemble Classifier may be the superior model, descriptive statistics derived from the confusion matrices indicate that the AdaBoost with CST achieves significantly higher accuracy.

**Table 2:** Measures of performance of exacerbation prediction models.

Model	Sensitivity	Specificity	PPV	NPV	Accuracy
EasyEnsemble Classifier	67.0 %	69.5 %	5.0 %	98.9 %	69.4 %
AdaBoost with CST	35.0 %	89.0 %	7.08 %	98.3 %	87.4 %

**Fig. 6:**
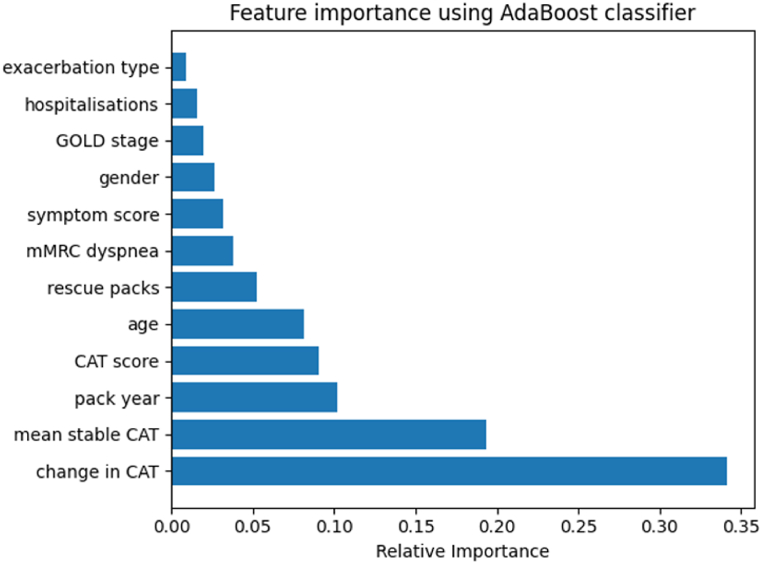
Feature importance computed from the AdaBoost with CST on the myCOPD dataset.

We used the built-in feature importance metric of AdaBoost to assess the frequency of feature use in its decision-making process. Feature importance is determined by the cumulative contribution of each feature across all weak classifiers in the ensemble. The higher the accumulated weight a feature has in the decision-making process of the ensemble, the more important it is considered. The relative feature importance of the AdaBoost with CST model is depicted in Fig. 6, revealing that CAT change, CAT mean, pack year, CAT score, and the number of rescue packs used in the last year are ranked with the highest relative importance. The prominence of the change in CAT score as the most important feature implies that the model is making timely predictions rather than relying solely on baseline characteristics for classification.

## 4 Discussion

An AdaBoost and an EasyEnsemble Classifier model were used to predict exacerbations using data extracted from a widely deployed digital therapeutic. When patient data was randomly split across training and test sets, AdaBoost with a CST and EasyEnsemble Classifier models achieved a sensitivity of 67 % and 35 % and a specificity of 69.5 % and 89 %, respectively.

### 4.1 The utility of patient-entered app data

Leveraging routinely patient-entered data from a real-world COPD self-management app, enabled a less burdensome and resource-intensive exacerbation prediction when contrasted with the demands of daily clinical monitoring and demonstrates similar performance to these more resource-intensive approaches. Xie et al. Measured daily physiological variables and their classifier for identifying patients at “high risk” or “low risk” of an exacerbation has an accuracy of 79.2 % compared to our best model with an accuracy of 87.8 % [20]. Mohktar and colleagues undertook patient classification and achieved a model accuracy of 71.8 % [21]. While these studies exhibit models with higher sensitivity, it is the elevated specificity of our model that ultimately contributes to its overall accuracy.

### 4.2 Labelling exacerbations: symptom-based vs. event-based

There is heterogeneity in the literature when it comes to labelling exacerbations. the choice of label relates to symptom-based or event-based definitions. Symptom-based labels have the advantage of capturing a larger number of self-reported exacerbations that can lead to models that are perhaps more useful for patient self-management. Event-based labels capture the most severe events which as a result may have variables that produce stronger signals prodromal to exacerbations resulting in better-performing predictive models. We believe using when patients report they have used their rescue pack or contacted primary healthcare or emergency services is an appropriate choice as this fit the clinical definition.

### 4.3 Data splitting strategies and algorithmic approach to class imbalance

We employed two distinct splitting strategies—by data and by participant. Unlike some prior approaches that generate models and validate them through methods like training on individuals before deploying on the same individuals or splitting randomly across the dataset [16,22,23], our choice to incorporate both as split by data and participants enables effective comparison and identification the model's ability to generalize to new unseen patient data. Opting not to split by patients may yield overly optimistic results, particularly in datasets like myCOPD, underscoring the necessity for a considerably larger patient pool to enhance inter-patient generalization.

We used two versions of the AdaBoost algorithm for forecasting exacerbations. our goal was to categorise patient records into either a stable state or an exacerbation state. However, the notable disparity in class sizes—where stable data points significantly outnumbered exacerbation data points—resulted in a pronounced imbalanced classification problem, with a dataset ratio of 1:44, signifying a moderate-to-severe imbalance. Imbalanced data poses challenges, particularly because predicting the minority class (exacerbation state) is more difficult due to its limited examples. Moreover, the surplus of cases from the majority class (stable state) can overshadow the minority class. Traditional classification algorithms, designed for roughly equal class distributions, exhibit bias toward the majority class, often neglecting the minority class. In the context of predicting COPD exacerbations, this bias can be a significant concern, as the minority class holds greater importance. Ensuring accurate modeling that mirrors real-world exacerbation patterns requires maintaining the current patient data distribution. Employing AdaBoost, an ensemble method, we made two key adaptations. The EasyEnsemble Classifier tackles imbalanced classes by random undersampling during training, prioritising sensitivity to exacerbations. The second adjustment involves a cost-sensitive tree, assigning higher weights to the class with a higher misclassification cost (exacerbation state).

### 4.4 Model performance and dynamic vs. fixed features

The specificity of the AdaBoost with CST is significantly higher (89.0 %) than the EasyEnsemble Classifier. The reason for the differing performance is likely due to the approaches selected for tackling the imbalanced classification problem. The EasyEnsemble Classifier uses an undersampling technique to balance the classes to improve model performance. Whilst the AdaBoost algorithm improves the scope of the data used for training the models, a significant amount of data from the majority class is still left unseen. This could have resulted in the specificity being lower than the AdaBoost with CST. However, the alternative cost-sensitive approach caused a significant drop in sensitivity. The weighting of the CST algorithm is designed to prevent the misclassification of the minority class. However, because the class imbalance is so large it seems that the majority class still overwhelms the training of the model. The myCOPD data consists of real-world data, so, there will likely always be a significant trade-off between sensitivity and specificity. Patients use the myCOPD app how they see fit and will modify their use based on their interpretations of the severity of their COPD.

The dataset employed for modelling in this study encompassed both dynamic (CAT score and symptom score) and fixed (age, gender, GOLD stage, etc.) features. We showed the most critical feature to the model was the change in the CAT score. This underscores the ability of the model to capture temporal fluctuations in the CAT score to enable the model to provide timely and accurate predictions.

### 4.5 Strengths and limitations

Key strengths of our study design include the duration of data collection, the large number of patients and the large number of exacerbations captured. This is one of the largest datasets to date indicating that the predictive models generated in this study are more generalisable to the wider population. Furthermore, generating models from an established and widely available digital therapeutic creates the potential to deploy an exacerbation support tool more widely providing a greater impact on patient care. Most studies monitor sample sizes for 3–6 months and as a result, only capture a handful of exacerbations [24,25]. This study's data set spans 4 years, with 506 participants and 1263 exacerbations captured. A notable limitation lies in the potential lack of external validity attributed to patients self-reporting exacerbations. However, it's essential to consider that this self-reporting method involves patients explicitly noting the use of their rescue medication, aligning directly with a critical aspect of the exacerbation definition. Our model uses symptom score, CAT score, and changes in CAT score as key features, incorporating valuable insights from patients about their health. However, relying on subjective measures introduces variability and subjectivity, impacting data consistency. Despite this limitation, the CAT stands out as a rigorously tested patient-reported outcome tool in COPD. It possesses extensive construct and external validity and is recognized by regulatory bodies as a crucial outcome measure in COPD, complete with a well-defined Mean Clinically Important Difference (MCID).

The key limitation of our prediction model lies in the suboptimal sensitivity of the Adaboost with CST, specifically at 35 %. This low sensitivity level may render it impractical for real-world exacerbation interventions. While our initial exploration indicates some predictive value in the features, the inherent complexity of real-world data presents a formidable challenge in accurately forecasting events. Similarly, the very low PPV of our model introduces additional challenges for its adoption in real-world scenarios. This low PPV is attributed to the high volume of stable state data. Comparatively, Patel et al. and their intervention COPDPredict™ has a greater PPV [22]. The system identifies COPD exacerbations at a median of 7 days before a clinician-defined episode. The researchers found that COPDPredict™ has a sensitivity of 97.9 %, a specificity of 84.0 %, a PPV of 38.4 % and an NPV of 99.8 %. However this did not account for regression to the mean. In addition, participants were required to use COPDPredict™ every day for 6 months. This involved uploading self-assessments daily using the COPDPredict™ app, measuring FEV1 using connected spirometers and recording C-reactive protein using finger-prick testing Despite this detailed monitoring the PPV for an exacerbation remains modest at 38.4 %. In comparison, our model, while less accurate, is less burdensome to the patient-user by relying solely on their occasional self-input for disease state management.

## 5 Conclusion

This study demonstrates the potential exacerbation predictive value of routine patient-entered data in a real-world digital therapeutic. While the accuracy of the models is still relatively low, the negative predictive value is high indicating potential utility in supporting patients when to avoid rescue packs unnecessarily. Further work is required to improve positive predictive performance. This could be through incorporating sensing technologies in co-design partnership with patients followed by further clinical studies to validate a digital early exacerbation warning system which results in improved clinical outcomes in COPD.

## Ethics and consent

Following registration with the myCOPD app, each user is assigned an anonymous encrypted user key to ensure the protection of identifiable patient data. Before gaining full access to the myCOPD app, users are required to agree to the Terms & Conditions of Use and Privacy Policy. Failure to agree will prevent users from proceeding further.

The my mhealth Limited Privacy Policy states, “By signing up to use our app(s), you are entering into a contractual agreement with us, allowing us to collect and use your data as outlined within this policy”. Within this policy, it is specified that “We will use your data to review and assess the quality of our service(s)”.

This study aimed to asses myCOPD data to evaluate its potential to predict COPD exacerbations. All patient data used in this study has undergone complete anonymisation to safeguard privacy and confidentiality.

## Funding statement

The author is supported by the 10.13039/501100000266EPSRC Digital Health and Care Centre for Doctoral Training (CDT) at the 10.13039/501100000883University of Bristol (10.13039/100014013UKRI Grant No. EP/S023704/1). This study was supported by the National Institute for Health and Care Research 10.13039/100015250Bristol Biomedical Research Centre. The views expressed are those of the authors and not necessarily those of the NIHR or the Department of Health and Social Care.

## Data availability statement

Data supporting this study cannot be made available due to commercial restrictions.

## CRediT authorship contribution statement

**Henry M.G. Glyde:** Writing – review & editing, Writing – original draft, Visualization, Validation, Methodology, Investigation, Data curation, Conceptualization. **Alison M. Blythin:** Writing – review & editing, Supervision, Resources, Project administration. **Tom M.A. Wilkinson:** Writing – review & editing, Supervision, Methodology, Conceptualization. **Ian T. Nabney:** Writing – review & editing, Supervision, Methodology, Conceptualization. **James W. Dodd:** Writing – review & editing, Supervision, Methodology, Investigation, Conceptualization.

## Declaration of competing interest

The authors declare the following financial interests/personal relationships which may be considered as potential competing interests. Tom Wilkinson reports a relationship with my mhealth ltd that includes: board membership. Alison Blythin reports a relationship with my mhealth ltd that includes: employment. If there are other authors, they declare that they have no known competing financial interests or personal relationships that could have appeared to influence the work reported in this paper.
