# Supplementary material for: Exacerbation predictive modelling using real-world data from the myCOPD app
Source: Heliyon. 2024 May 14;10(10):e31201. doi: 10.1016/j.heliyon.2024.e31201 (PMC11128912; doi:10.1016/j.heliyon.2024.e31201)
Supplement: Multimedia component 1 [file mmc1.docx]

SUPPLEMENT

- **The CAT score:** Derived from the COPD Assessment Test, is a validated health status tool using 8 graded questions to assess the impact of COPD on daily living. Users are prompted to complete the CAT monthly but can do so as frequently as they prefer within the app.
- **Mean stable CAT:** Derived from the CAT score, this feature is generated by calculating the average CAT score when users reported their symptoms as "normal for me”.
- **Change in CAT:** Determined by subtracting the CAT mean from their specific CAT score for that instance.
- **Symptom score:** A 4-point variable reflecting the patient's daily well-being, and users are encouraged to complete it daily. Notably, a rise in the symptom score from 1 to 2 before an exacerbation may indicate the use of a reliever inhaler, suggesting a decline in symptoms and the potential onset of an exacerbation. Age is recorded as the individual's age at the time of data entry, and gender is self-reported as male, female, or not specified. Fort further details on dynamic and stable variables see supplement.
- **Age:** Recorded as the individual's age at the time of data entry.
- **Gender:** Self-reported as male, female, or not specified.
- **GOLD stage:** Categorises COPD severity based on FEV1 and symptoms/exacerbation history.
- **mMRC dyspnea scale:** An assessment of breathlessness, graded from 0 to 4 based on increasing levels of difficulty during various activities.
- **Pack Year:** Quantifies cumulative cigarette smoke exposure, calculated by multiplying packs smoked per day by years smoked.
- **Rescue packs:** The instances in which a patient self-administered antibiotics and/or corticosteroids for exacerbation management for that year.
- **Hospitalisations:** The number of admissions for AECOPD for that year.
- **Exacerbation type:** Reflects the severity of the last exacerbation, categorized as moderate (treated at home) or severe (requiring hospital or emergency department admission).
